# Pure Small Cell Carcinoma of the Bladder: A Case Report

**DOI:** 10.4021/wjon2010.06.210w

**Published:** 2010-05-19

**Authors:** Amel Trabelsi, Soumaya Ben Abdelkrim, Samah Tebra, Olfa Gharbi, Lilia Jaidane, Noureddine Bouaouina, Dajla Bakir Abbassi, Moncef Mokni

**Affiliations:** a Department of Pathology, Farhat Hached Hospital, Sousse, Tunisia; b Department of Radiotherapy, Farhat Hached Hospital, Sousse, Tunisia; c Department of Medical Oncology, Farhat Hached Hospital, Sousse, Tunisia; d Department of Radiology, Farhat Hached Hospital, Sousse, Tunisia

**Keywords:** Urinary bladder, Small cell carcinoma, Pathology

## Abstract

Small cell carcinoma of the urinary bladder is an uncommon tumor that has been described in case reports or small series. We report a new case in a 67-year-old male who presented with gross hematuria and irritative symptoms. Cystoscopy revealed an extensive mass of the bladder and computed tomography scan showed an important thickening of the bladder wall. Diagnosis of small cell carcinoma was established after radical cystectomy and microscopic examination. The patient received pelvic hemostatic radiotherapy and platinium-based chemotherapy. Three months after the diagnosis, he developed bone, renal and adrenal metastases.

## Introduction

Small cell carcinoma (SCC) of the urinary bladder is distinctly uncommon, accounting for less than 1% of all bladder cancers [1]. Unlike urothelial carcinoma, SCC of the bladder behaves aggressively like its pulmonary counterpart. We report a new case in a 67-year-old male treated by surgery, radiotherapy and chemotherapy and we present a brief review of the literature.

## Case Report

**Figure 1:**
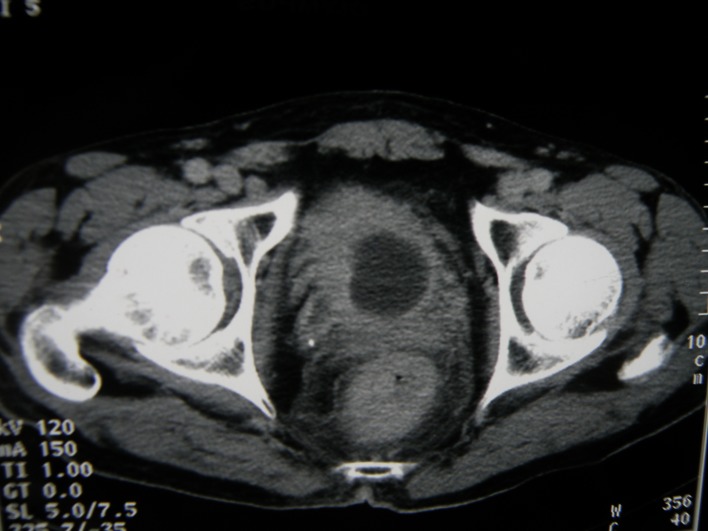
Computed tomography scan showing an important thickening of the bladder wall.

**Figure 2:**
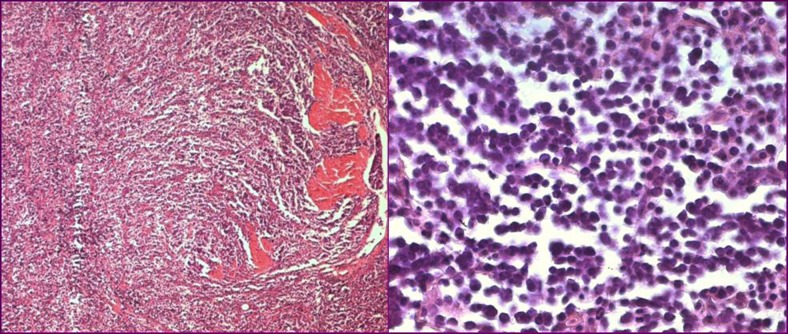
Histological sections showing a poorly differentiated carcinoma infiltrating the muscle (left, original magnification x 40) composed of sheets of uniformly small, round cells with overlapping nuclei lacking prominent nucleoli (right, original magnification x 400).

**Figure 3:**
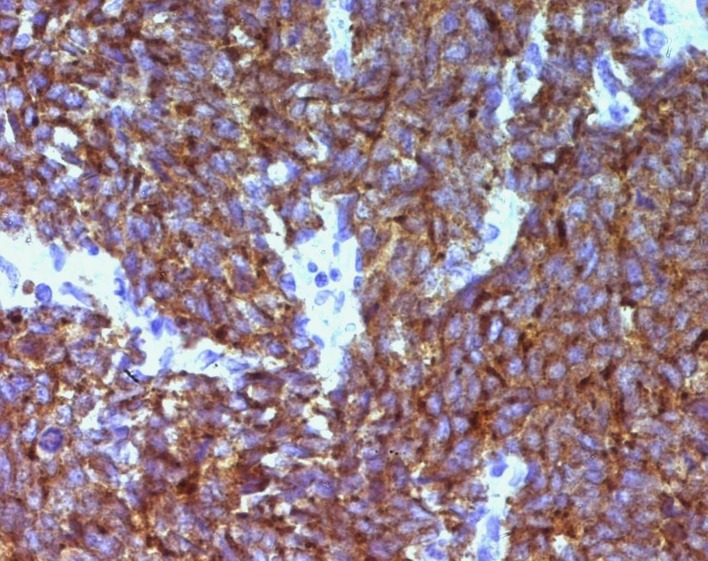
Synaptophysin immunostaining in SCC of the bladder (immunohistochemistry x 400).

A 67-year-old man presented with gross hematuria and irritative urinary symptoms. He had a 35-pack year history of smoking and no family history of genitourinary malignancy. Cystoscopy revealed an extensive polypoid mass of the bladder. Computed tomography scan showed an extensive thickening of the bladder wall with extension to the perivesical fat (Fig. 1). Pathologic examination of the surgical specimens of transurethral resection was consistent with the diagnosis of poorly differentiated carcinoma infiltrating the muscle and radical cystoprostatectomy with bilateral lymph node dissection was performed. Grossly, there was a 4 x 4 x 2 cm polypoid solid mass in the dome with an important thickening of the bladder wall. Microscopically, the tumor was composed of sheets of uniformly small, round, mitotically active cells with overlapping nuclei lacking prominent nucleoli (Fig. 2). Areas of necrosis were found. Immunohistochemical stains showed immunoreactivity of most of tumor cells for synaptophysin (Fig. 3) and neuron specific enolase. On the basis of these morphological and immunohistochemical findings, he was diagnosed of primary pure SCC of the bladder. The tumor invaded the muscle and the perivesical fat. Computed tomography of the lung and bone were normal. There were no lymph nodes metastases. The patient received thirty cycles of pelvic hemostatic radiotherapy and eight cycles of platinium-based chemotherapy. Three months later, he developed bone, renal and adrenal metastases.

## Discussion

SCC of the urinary bladder is a rare disease that accounts for less than 1% of all cancers [1]. It was initially described in 1981 by Cramer et al. [2]. Since then, less than 900 cases have been reported [3]. Gross hematuria is the most common symptom and most patients are men with history of smoking. Pathogenesis is uncertain; however the multipotent stem cell theory applies best to this tumor [4]. Histologically, SCC of the bladder is indistinguishable from its pulmonary counterpart. The diagnosis is based on the criteria established by the WHO classification system. The tumor is composed of sheets of uniformly small, round, mitotically active cells with overlapping nuclei and evenly distributed chromatin, lacking prominent nucleoli; tumor necrosis and crush artifact are commonly seen [3]. Immunohistochemical staining for the markers of endocrine differentiation including synaptophysin, chromogranin and NCAM can help in the diagnosis, particularly on transurethral resection material [3, 5]. Pure SCC of the bladder is an unusual malignancy and most of these tumors were mixed with urothelial cell carcinoma [1, 3]. Seeing that the presence of SCC portends a poor outcome, it seems appropriate to classify the tumors with mixed histological patterns as SCC rather than urothelial carcinoma with neuroendocrine features [1]. The optimal treatment strategy for bladder SCC is still unclear [6], but the combination of surgery, radiotherapy and chemotherapy is presently used. Radical cystectomy alone may be used only in small volume disease. Nevertheless, chemotherapy plays a prominent role in the management of these tumors [3]. Like SCC of the lung, Cisplatine-Etoposide regimen is usually used in SCC of the bladder [6, 7]. Radiotherapy can be performed to palliate brain metastases and symptomatic bone metastases [3]. Recently, the use of chemotherapy and radiotherapy with bladder preservation protocol seems to be an attractive concept [6]. SCC of the bladder is highly aggressive, mostly diagnosed at advanced stage. The pure form appears to have a poorer outcome than the mixed form; but combination of local treatment and chemotherapy seems to confer a more favorable prognosis [1]. In conclusion, the prognosis of SCC of the bladder remains poor; improvement of patients' outcome may depend on the identification of new molecular markers that could be used in early diagnosis and in novel targeted therapies [1].
